# Pathogenesis of *Porphyromonas gingivalis* peptidylarginine deiminase: from periodontal microenvironment to systemic diseases

**DOI:** 10.3389/fcimb.2026.1895458

**Published:** 2026-08-20

**Authors:** Weifan Shao, Jiahui Li, Yeting Wang, Zi’ang Qiao, Jiayou Li, Jingbo Liu

**Affiliations:** Department of Periodontics, School of Stomatology, China Medical University, Shenyang, China

**Keywords:** citrullination, outer membrane vesicles, peptidylarginine deiminase, *Porphyromonas gingivalis*, systemic diseases

## Abstract

Periodontitis is a globally prevalent chronic inflammatory disease that exerts profound effects on both oral and systemic health. *Porphyromonas gingivalis* is a key pathogenic bacterium in periodontitis. Its secreted peptidylarginine deiminase (PPAD) is a unique protein-citrullinating enzyme. It catalyzes the citrullination of arginine residues in proteins, thereby altering protein structure and function. This review systematically integrates current evidence to elucidate the enzymatic properties, secretion regulatory mechanisms, and pathogenic roles of PPAD in the periodontal microenvironment and distant organs. PPAD serves as a critical driver of periodontitis progression. It also acts as a molecular bridge linking oral infection to remote pathologies. This bridging effect is achieved through outer membrane vesicle-mediated systemic dissemination. PPAD-mediated citrullination constitutes a central nexus connecting this chronic oral infection with various comorbidities, providing a theoretical basis for the development of PPAD-targeted anti-virulence therapeutic strategies against periodontitis and its associated systemic conditions.

## 1 Introduction

Periodontitis is a prevalent chronic inflammatory disease of the oral cavity, which has been associated with various systemic diseases (Hajishengallis, 2022). According to the 2019 Global Burden of Disease Study, approximately 1.1 billion people worldwide suffer from periodontal diseases of varying severity, with over 530 million cases of severe periodontitis (Chen et al., 2021). This condition exerts a profound impact on human quality of life. It represents not only a significant threat to oral health but has also been linked to multiple remote pathologies, although direct causal relationship remain to be established for most of these conditions. In recent years, the “oral-systemic interaction” paradigm has prompted growing research interest in dentistry and immunology, focusing on potential pathways through which periodontal pathogens might influence distant organ pathologies.

*Porphyromonas gingivalis* peptidylarginine deiminase (PPAD) is one of the virulence factors of *Porphyromonas gingivalis* (*P. gingivalis*). Its homologs are also present in other Porphyromonas species, such as *Porphyromonas gulae* and *Porphyromonas loveana*. However, these homologs are primarily derived from non-human hosts. Due to its unique citrullinating activity, PPAD is emerging as a critical molecular link between periodontal infection and systemic pathologies (Wegner et al., 2010; Potempa et al., 2017). Unlike mammalian PADs, which require neutral pH and calcium for activity, PPAD functions optimally under acidic conditions and exhibits strict specificity for c-terminal arginine residues. Anchored to the outer membrane of *P. gingivalis* via the type IX secretion system (T9SS), PPAD is packaged into and released via outer membrane vesicles (OMVs). In murine models, the lipid bilayer of OMVs effectively protects PPAD from extracellular proteolytic degradation. This provides the structural basis for its systemic delivery into the circulation in a catalytically competent form. It also suggests the potential of PPAD to promote aberrant citrullination in distant organs (Gabarrini et al., 2018a). This “Trojan horse”-like delivery mechanism allows PPAD to introduce citrullinated neoepitopes in sites such as the synovial membrane, vascular walls, and intestinal mucosa. These neoepitopes repeatedly stimulate immune responses and break immune tolerance. As a result, PPAD becomes a potential key link driving various systemic diseases, including rheumatoid arthritis, atherosclerosis, and inflammatory bowel disease.

However, the pathogenic mechanisms of PPAD remain incompletely understood. Although substantial evidence indicates that PPAD can citrullinate multiple RA-associated autoantigens and drive the generation of anti-citrullinated protein antibodies (ACPAs) (Liu et al., 2022), the specific molecular pathways through which PPAD contributes to individual systemic conditions, its complex interplay with the host immune system, and its feasibility as a therapeutic target remain to be thoroughly elucidated. In this context, systematically characterizing the biological properties of PPAD and its “local-to-remote” dissemination pathway will not only facilitate deciphering the pathogenesis of this oral condition but also provide a theoretical framework for developing PPAD-targeted interventions against related comorbidities. This review summarizes the enzymatic features and secretion-regulatory mechanisms of PPAD, elaborates on OMV-mediated systemic delivery and the underlying mechanisms conferring resistance to host proteases, and focuses on dissecting its pathogenic paradigms in conditions including periodontitis, rheumatoid arthritis, atherosclerosis, and inflammatory bowel disease. Finally, we discuss PPAD-targeted intervention strategies and translational prospects, aiming to provide novel insights into interrupting the local-to-systemic disease axis.

**Figure 1:**
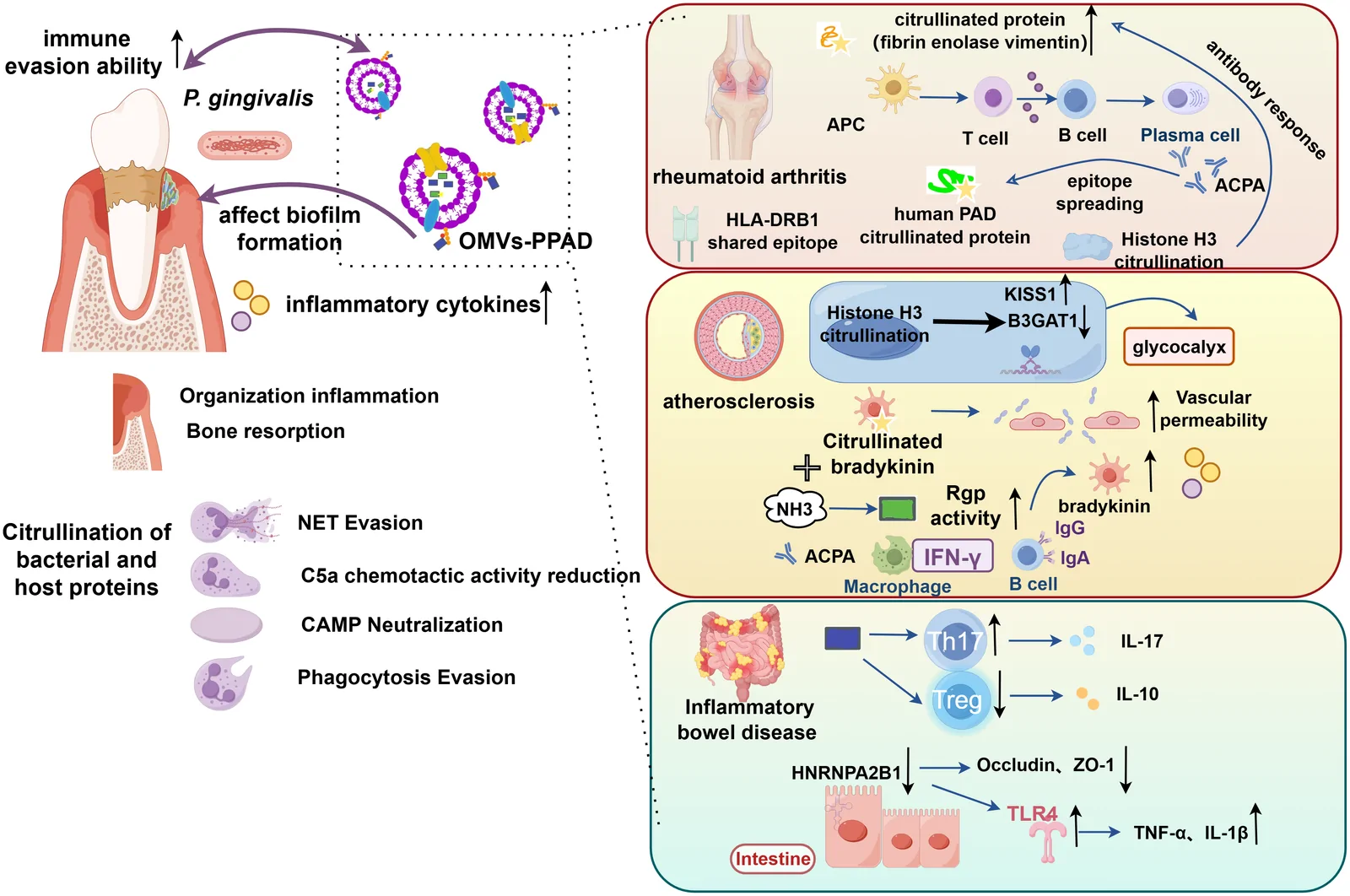
Schematic illustration of PPAD-mediated local periodontal pathogenesis and systemic dissemination. Left panel: In the periodontal pocket, PPAD citrullinates bacterial and host proteins to subvert immune defenses (C5a inactivation, NET evasion, phagocytosis resistance, and CAMP neutralization), modulate biofilm dynamics, amplify inflammation (PGE_2_, IL-36γ, Th17 skewing), and promote tissue destruction and bone resorption. Right panel: OMV-encapsulated PPAD enters systemic circulation and contributes to rheumatoid arthritis (citrullinated neoepitope generation and ACPA production), atherosclerosis (endothelial glycocalyx injury via CitH3/B3GAT1, vascular permeability enhancement), and inflammatory bowel disease (intestinal barrier disruption via HNRNPA2B1 downregulation and Th17/Treg imbalance). Dashed arrows indicate the OMV-mediated dissemination route from periodontal tissues to distant organs. By Figdraw.

As outlined above, PPAD operates through a dualistic pathogenic paradigm: it remodels the periodontal microenvironment locally while disseminating systemically via OMVs to drive pathology in distant organs. **Figure 1** provides an integrated schematic of this “local–systemic” axis, summarizing the local virulence network and the downstream mechanisms in rheumatoid arthritis, atherosclerosis, and inflammatory bowel disease. The following sections dissect each component of this pathway in detail.

## 2 Biological characteristics of PPAD

### 2.1 Distinctive catalytic properties of PPAD

In 1981, Fujisaki et al. first discovered PPAD, a protein-modifying enzyme that is one of the important virulence factors of *P. gingivalis (*Fujisaki and Sugawara, 1981). Mammalian peptidylarginine deiminase (PAD) is a calcium-dependent enzyme that catalyzes the conversion of arginine residues to citrulline and plays a crucial role in the citrullination process (Zhang et al., 2021). Although PPAD shares similar biochemical characteristics with PAD and is considered a functional analog of PAD, the two enzymes differ in gene structure and exhibit low sequence identity (Catalan et al., 2024), with no genetic relatedness (Montgomery et al., 2016). In addition, PPAD and PAD also possess distinct substrate specificities. PPAD preferentially targets C-terminal arginine residues and remains stable in a low-pH environment, whereas PAD primarily modifies arginine residues located within the peptide chain.

Since its discovery, PPAD has been biochemically and structurally characterized. PPAD was initially purified by (McGraw et al., 1999), and subsequently cloned by Rodriguez et al (Rodríguez et al., 2009). In 2015, Goulas et al. determined the three-dimensional structure of PPAD, which comprises four domains: an N-terminal signal peptide, a catalytic domain, an immunoglobulin-like fold domain, and a C-terminal domain (Goulas et al., 2015). Subsequent studies have shown that PPAD is recognized by T9SS through its C-terminal domain and translocated to the outer membrane (OM), where it is anchored via post-translational modification with A-type lipopolysaccharide (A-LPS) and subsequently enriched in OMVs through outer membrane blebbing; this modification is essential for its stable retention in the OM and OMVs (Gabarrini et al., 2018a). Furthermore, the N-terminally truncated form of PPAD present under native conditions exhibits higher catalytic activity and is protected from autocitrullination-induced inactivation (Konig et al., 2015).

### 2.2 Protective secretion mediated by the T9SS–OMV axis

**Figure 2:**
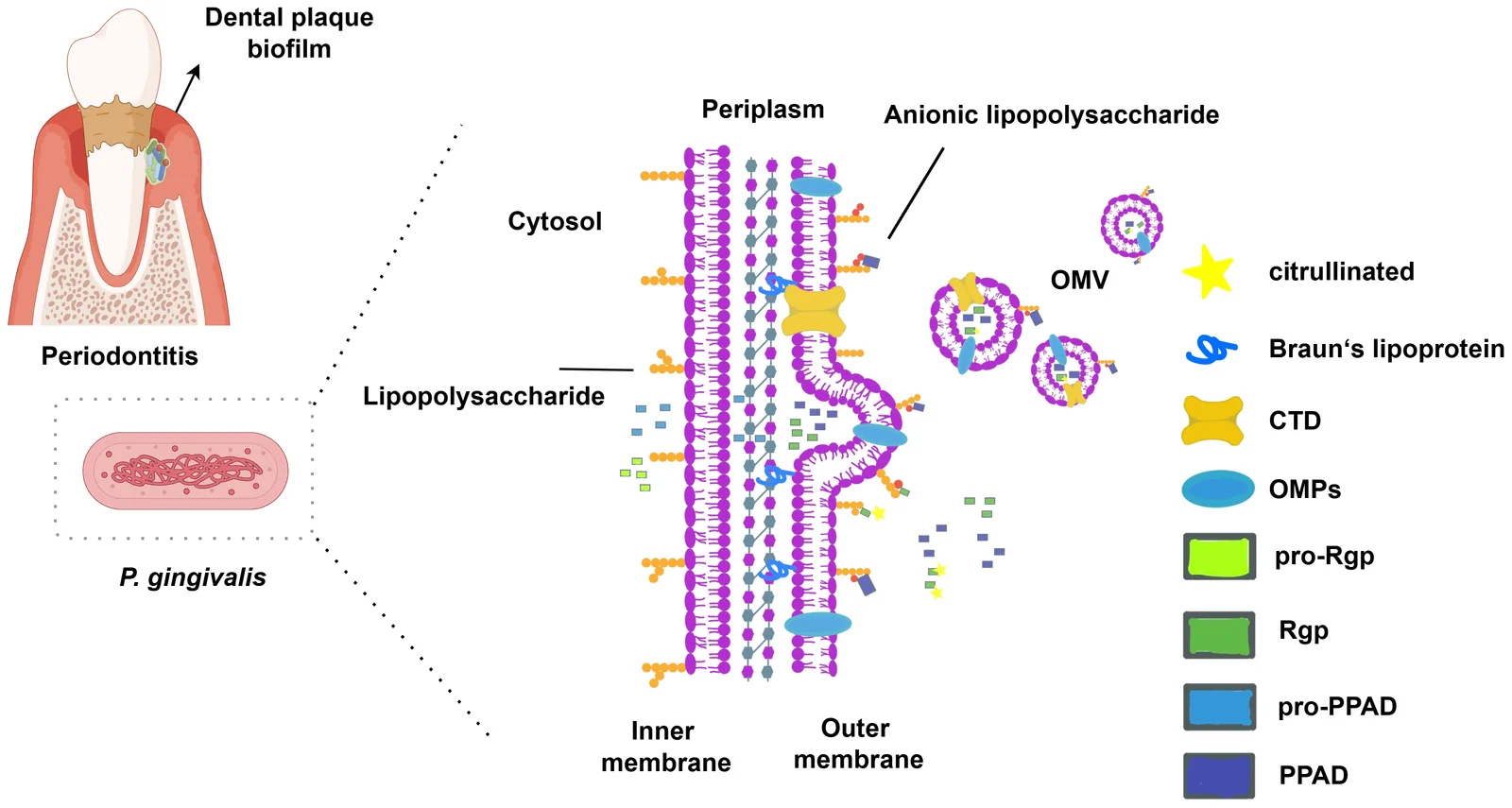
Molecular mechanism of T9SS-dependent secretion and OMV packaging of PPAD and Rgp in *Porphyromonas gingivalis.* Left: *P. gingivalis* colonizes the dental plaque biofilm within the periodontal pocket, establishing the local niche for virulence factor production. Right: Enlarged schematic of the *P. gingivalis* cell envelope showing the T9SS-mediated translocation pathway. PPAD and Rgp precursors (pro-PPAD and pro-Rgp) are transported across the inner membrane into the periplasm, where their C-terminal domains (CTDs) are recognized by the T9SS apparatus. The PorU sortase cleaves the CTD and covalently links the cargo to A-LPS on the outer membrane. Upon localized membrane blebbing, A-LPS-anchored PPAD and Rgp are captured and packaged into outer membrane vesicles (OMVs). Yellow stars indicate citrullinated substrates/products. Braun’s lipoprotein and outer membrane proteins (OMPs) are also depicted for structural context. By Figdraw.

PPAD secretion and localization follow a sophisticated “protective secretion” program that ensures the enzyme maintains catalytic activity at both local and systemic sites. First, the T9SS governs transmembrane transport: the PorL/PorM complex on the inner membrane harnesses proton motive force to generate a rotary motor, driving the rotation of the PorK/PorN ring beneath the outer membrane. After cargo traverses the outer membrane via the Sov (SprA) transporter, the outer membrane shuttle protein PorV collects and delivers it to the cell-surface attachment complex. Finally, the PorU sortase cleaves the C-terminal domain of PPAD and covalently attaches its newly exposed C-terminus to A-LPS, thereby anchoring the protein to the outer membrane. As A-LPS is an integral constituent of the outer membrane lipid bilayer, this covalent attachment incorporates PPAD into the membrane; accordingly, when localized membrane regions undergo blebbing to form OMVs, the anchored PPAD is physically captured and packaged into the budding vesicles. This multi-step secretion and vesiculation program is schematically illustrated in **Figure 2**. As depicted, PPAD and Rgp precursors are translocated across the inner membrane and subsequently recognized by the T9SS machinery at the outer membrane. Following PorU sortase-mediated cleavage of the C-terminal domain (CTD), both enzymes are covalently attached to A-LPS and enriched in localized outer membrane regions that undergo blebbing to form OMVs. This structural organization ensures that PPAD and Rgp are co-packaged within the same vesicular carriers, providing the physical basis for their synergistic citrullination cascade at both local and systemic sites. Studies of clinical isolates have revealed two molecular forms of PPAD: a membrane-bound form (~75–85 kDa) that is anchored via A-LPS and enriched in OMVs. The second arises when A-LPS modification is obstructed, T9SS processing yields a ~47 kDa soluble PPAD accompanied by minor high-molecular-weight aggregates (Gabarrini et al., 2018a). This divergence carries dual biological significance. First, within the periodontal pocket, A-LPS anchoring and encapsulation by the OMV lipid bilayer shield PPAD from degradation by host proteases and acidic environments, preserving local citrullination activity. Upon entering the circulation, OMV-carried PPAD retains enzymatic activity and can be transported with vesicles to distant sites such as joints and vascular walls. *In vitro* experiments demonstrate that OMVs can be internalized by neutrophils and macrophages, and the carried PPAD may be released intracellularly to modify host proteins, whereas soluble PPAD primarily acts on extracellular substrates (Curtis et al., 2025). Second, the A-LPS dependent anchoring of PPAD is integral to the integrity of border T9SS secretory program and outer membrane dynamics. PPAD deficiency causes gingipain-derived adhesion proteins to be retained in the biofilm matrix, reduces Rgp activity in the supernatant, and is accompanied by decreased OMV release (Vermilyea et al., 2021). These findings indicate that PPAD is not merely a passenger cargo of the T9SS but actively contributes to maintaining the functional architecture of the outer membrane and the efficiency of vesicle budding, thereby influencing the delivery of multiple virulence factors beyond PPAD itself.

### 2.3 Synergy with arginine-specific gingipains: the citrullination cascade

**Figure 3:**
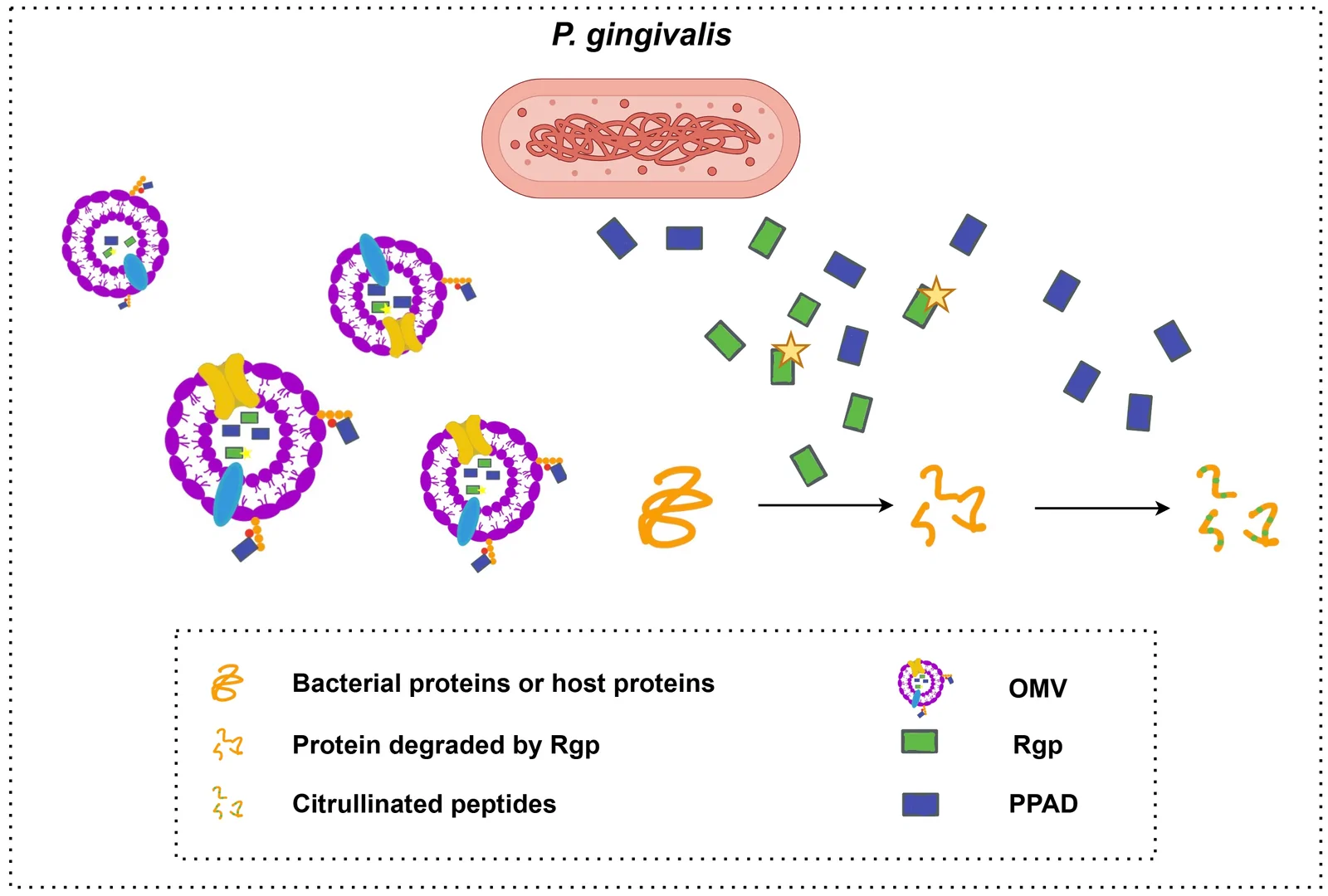
Schematic illustration of the synergistic citrullination cascade mediated by Rgp and PPAD. Arginine-specific gingipain (Rgp, green) proteolytically cleaves bacterial and host proteins to expose C-terminal arginine residues. These newly generated termini serve as substrates for PPAD (blue), which catalyzes their conversion to citrulline (yellow stars), producing citrullinated peptides. This sequential reaction constitutes the core of the *P. gingivalis* citrullinome. By Figdraw.

PPAD-mediated citrullination requires synergistic cooperation with arginine-specific gingipains (Rgps). Gingipains are a family of cysteins proteases secreted by *P. gingivalis*, comprising two arginine-specific isoforms (RgpA and RgpB) and one lysine-specific gingipain (Kgp) (Guo et al., 2010). These proteases constitute major virulence factors of *P. gingivalis*, contributing to nutrient acquisition, host tissue degradation, evasion of innate immunity, and modulation of the inflammatory response. Rgps initially cleave both bacterial and host proteins, exposing C-terminal arginine residues that are subsequently deiminated by PPAD to generate C-terminal citrullinated peptides. In Rgp-deficient *P. gingivalis* strains, overall protein citrullination is markedly reduced (Montgomery et al., 2016). This sequential enzymatic cascade is schematically illustrated in **Figure 3**. As shown, Rgp initially cleaves bacterial or host proteins to expose C-terminal arginine residues, which are subsequently deiminated by PPAD to generate citrullinated peptides. This coordinated proteolytic and post-translational modification sequence constitutes the molecular foundation of the *P. gingivalis* citrullinome. Furthermore, citrullination of Rgp by PPAD protects the protease from autocleavage at arginine residues, thereby preserving its sustained capacity to generate peptides bearing C-terminal arginine. This bidirectional interplay-wherein Rgps supply substrates for PPAD while PPAD, in turn, stabilizes Rgp activity-exemplifies a mutualistic enzymatic relationship that is central to the citrullinome of *P. gingivalis*. This “enzyme protection–enzyme stabilization” cascade represents a sophisticated strategy by which *P. gingivalis* subverts host immune defenses (Stobernack et al., 2018).

### 2.4 Multifaceted inhibition of host innate immune defenses

Studies have demonstrated that PPAD attenuates host innate immune defenses through multiple mechanisms. Bielecka et al. showed that PPAD-mediated citrullination of C5a markedly diminishes its chemotactic activity toward neutrophils and attenuates C5a receptor-mediated calcium influx signaling, thereby suppressing complement C5a function (Bielecka et al., 2014). Stobernack et al. reported that PPAD-deficient *P. gingivalis* exhibits significantly enhanced neutrophil binding and internalization, indicating that PPAD restricts bacterial phagocytosis and intracellular uptake by neutrophils (Gabarrini et al., 2018b). Furthermore, upon encountering neutrophil extracellular traps (NETs) that have already been released, PPAD citrullinates extracellular NET-associated histone H3, a modification that appears to weaken NET structure and reduce its bactericidal activity, thereby facilitating *P. gingivalis* evasion (Vitkov et al., 2018). This mechanism is distinct from NETosis itself, in which host PAD4 drives intracellular histone hypercitrullination to promote chromatin decondensation and NET release. Additionally, PPAD can citrullinate the cationic antimicrobial peptide LP9 derived from lysozyme, abrogating its membrane-disrupting capacity and bactericidal activity (Stobernack et al., 2018).

## 3 Local pathogenic mechanisms of PPAD: modulation of the periodontal microenvironment

Periodontitis is an inflammatory disease driven by a complex, dysbiotic oral microbiota. Citrullination is detectable across diverse inflammatory tissues, and this process is inflammation-dependent (Makrygiannakis et al., 2006). Studies have demonstrated increased expression of citrullinated proteins in gingival tissues of periodontitis patients, together with seropositivity for ACPAs. Compared with ACPA-negative rheumatoid arthritis (RA) patients, ACPA-positive individuals exhibit a significantly higher prevalence of moderate-to-severe periodontitis (Dissick et al., 2010). Furthermore, ACPA levels are positively correlated with tooth loss attributable to periodontitis, Community Periodontal Index, and Clinical Attachment Loss (Karkowska-Kuleta et al., 2018). PPAD may contribute to periodontitis pathogenesis by promoting inflammatory responses, modulating biofilm formation, and enhancing the immune evasion capacity of *P. gingivalis* through citrullination.

### 3.1 Genetic determinants of virulence variation

PPAD genetic polymorphisms significantly contribute to inter-strain virulence heterogeneity of *P. gingivalis*. Bereta et al. cloned and identified a novel hyperactive variant (PPAD-T2) from clinical isolates, which harbors a G231N/E232T/N235D triple amino acid substitution in its catalytic domain (Bereta et al., 2019). This variant exhibits higher substrate conversion efficiency compared with the wild type, albeit with attenuated substrate binding affinity. Cohort studies have confirmed that strains carrying the PPAD-T2 variant are detected in approximately 30% of chronic periodontitis patients and are associated with more severe clinical attachment loss and probing depth (Bereta et al., 2024). However, Kamińska et al. reported that PPAD-T2 did not significantly influence conventional clinical periodontal parameters, including bleeding on probing, clinical attachment loss, and probing depth, but substantially reshaped salivary microbiota composition (Strzelec et al., 2025). The discrepant findings between these two studies may be attributed to differences in sample size, population heterogeneity, or detection methodologies, suggesting that the pathogenic mechanism of PPAD-T2 may involve microbiota modulation rather than direct association with traditional clinical indices, which warrants further validation.

### 3.2 PPAD-mediated regulation of biofilm dynamics

PPAD significantly enhances the adherence of *P. gingivalis* to *Candida albicans* cells. As an obligate anaerobe, *P. gingivalis* benefits from its association with *Candida albicans*-a facultative anaerobe that consumes oxygen and thereby reduces local oxygen levels-allowing *P. gingivalis* to survive under otherwise aerobic conditions (Karkowska-Kuleta et al., 2018). The regulatory role of PPAD in *P. gingivalis* biofilm formation exhibits strain-dependent variation. In the ATCC 33277 strain, PPAD deficiency does not impair monospecies biofilm development (Aliko et al., 2018). In contrast, PPAD deletion in the *P. gingivalis* 381 strain markedly promotes biofilm formation, yielding mature biofilms with increased extracellular matrix and abundant protein aggregates (Vermilyea et al., 2019). Mechanistically, PPAD may citrullinate T9SS cargo proteins, particularly gingipain-derived adhesins, thereby restricting their accumulation within the biofilm matrix and constraining excessive biofilm growth (Vermilyea et al., 2019). Additionally, PPAD is significantly upregulated during bacterial surface translocation, where it promotes OMV biogenesis and release, enhances Rgp-mediated proteolytic activity, and drives the transition from sessile growth to a surface-migratory lifestyle (Vermilyea et al., 2021). PPAD deficiency alters arginine metabolism, resulting in spermidine accumulation and reduced OMV production, which delays surface translocation while concomitantly enhancing biofilm formation. Furthermore, PPAD-mediated fibrinogen citrullination may disrupt its inhibitory effect on bacterial coaggregation, thereby contributing to biofilm stabilization and infection dissemination (Jenning et al., 2020). Collectively, these findings indicate that PPAD serves as a critical regulator of *P. gingivalis* biofilm development, with its functional impact contingent upon strain genetic background and environmental conditions.

### 3.3 Direct involvement in tissue destruction and inflammatory regulation

#### 3.3.1 PPAD-mediated direct tissue destruction and barrier dysfunction

At the epithelial barrier level, PPAD citrullinates epidermal growth factor, resulting in defective cell cycle regulation. Through suppression of suppressor of cytokine signaling 3 and interferon regulatory factor 1 signaling, PPAD inhibits epithelial cell proliferation and migration, compromises local protective epithelium, delays periodontal tissue healing, and exacerbates tissue damage (Pyrc et al., 2013). Gawron et al. demonstrated that PPAD activity facilitates *P. gingivalis* adhesion to and invasion of fibroblasts (Gawron et al., 2014). Furthermore, by citrullinating *P. gingivalis* surface proteins, PPAD activates the prostaglandin E_2_ (PGE_2_) signaling pathway in fibroblasts, manifesting as elevated expression of cyclooxygenase-2 and microsomal prostaglandin E_2_ synthase-1, significantly enhanced PGE_2_ secretion, and consequent induction of osteoclast genesis, ultimately leading to alveolar bone loss. However, PPAD inactivation or deletion does not affect *P. gingivalis* adhesion to or invasion of gingival epithelial cells (Aliko et al., 2018); this discrepancy may be attributed to differences in integrin expression profiles on distinct cell surfaces.

Furthermore, ammonia generated during PPAD-catalyzed citrullination may exert dual functions in the acidic oral microenvironment: on the one hand, neutralizing acidic conditions to protect the survival of *P. gingivalis* (Konig et al., 2015); on the other hand, directly suppressing neutrophil function, thereby indirectly facilitating sustained bacterial destruction of local tissues (Shawcross et al., 2008).

#### 3.3.2 PPAD-driven inflammatory response and immune evasion

Regarding inflammatory signaling pathways and cytokine networks, in a co-culture model of *P. gingivalis*, naive human dental follicle stem cells, and neutrophils, PPAD upregulates the JNK/ERK pathway to prolong neutrophil survival and enhance their migratory capacity (Gawron et al., 2014). Mechanistically, PPAD suppresses neutrophil apoptosis by upregulating the expression of anti-apoptotic proteins A1/Bfl-1 and Bcl-xL, while concurrently promoting the secretion of IL-6 and TNF-α. Compared with the wild-type *P. gingivalis* strain, PPAD gene-deficient strains downregulate the expression of TNF-α, IL-1β, IL-6, IL-10, and monocyte chemoattractant protein-1 genes upon stimulation of human monocytes (Karkowska-Kuleta et al., 2018), indicating that PPAD serves as a critical amplifier of pro-inflammatory responses. In gingival epithelial cells, PPAD significantly upregulates the expression of multiple pro-inflammatory genes, including IL-36γ, IL-1β, CCL20, and CXCL8 (Aliko et al., 2018). IL-36γ and IL-1β belong to the IL-1 superfamily and function as potent immunomodulators (Makrygiannakis et al., 2006), whereas CCL20 and CXCL8 are chemokines that orchestrate neutrophil and T-cell recruitment. Among them, IL-36γ is one of the most robustly induced inflammatory genes following *P. gingivalis* infection of oral epithelial cells; it not only directly promotes inflammatory responses but also further amplifies the expression of CXCL8 and CCL20, thereby generating a Th17-biased inflammatory microenvironment—a hallmark characteristic of chronic periodontitis lesions (Huynh et al., 2016). Concurrently, PPAD contributes to protecting *P. gingivalis* from phagocytic killing and suppresses the release of reactive oxygen species from neutrophils (Kriebel et al., 2018). By promoting neutrophil survival, inducing pro-inflammatory cytokine secretion, and simultaneously enhancing bacterial resistance to phagocytosis, this enzyme impedes inflammatory resolution, perpetuates the chronic state of periodontitis, and enables persistent infection within the tooth-supporting tissues (Prucsi et al., 2023).

With respect to pattern recognition receptor co-activation, host cells primarily recognize *P. gingivalis* through Toll-like receptor 2 (TLR2). PPAD activity and the expression of major fimbriae (FimA) constitute prerequisites for the activation of TLR2-dependent host signaling. PPAD-mediated citrullination of fimbrial components serves as the structural basis for FimA-TLR2 hyperactivation, which synergizes with PPAD activity to collectively upregulate downstream pro-inflammatory mediators, including TNF-α, IL-1β, mPGES-1, and COX-2 (Wielento et al., 2022).

#### 3.3.3 Integration of PPAD’s dualistic pathogenic roles: from local persistence to systemic autoimmunity

The apparently contradictory characteristics of PPAD—simultaneously suppressing acute immune defenses while amplifying chronic inflammation and dismantling immune tolerance—can be reconciled through a temporally and spatially coordinated pathogenic strategy (**Table 1**). Locally, PPAD-mediated immune evasion and ROS suppression secure a survival niche for *P. gingivalis*, allowing sustained enzymatic activity. Concurrently, PPAD remodels the periodontal microenvironment into a chronically inflamed, Th17-biased state. Systemically, OMV-encapsulated PPAD delivers citrullinated neoepitopes to distant organs, where immune tolerance is broken and autoimmune responses are initiated. Thus, immune evasion serves as the prerequisite for persistence, whereas pro-inflammatory citrullination and tolerance disruption constitute the pathogenic consequences of that persistence.

**Table 1:** The dualistic pathogenic role of PPAD: integrating immune evasion, ROS modulation, pro-inflammatory amplification, and immune tolerance breakdown.

Function	Molecular target/Substrate	Cellular and molecular mechanisms	Local (periodontal) consequences	Systemic (distant organ) consequences	Integrative pathogenic interpretation	References
Immune evasion	Complement C5a	Citrullination abolishes chemotactic activity toward neutrophils and attenuates C5a receptor-mediated calcium influx	Impairs complement-driven neutrophil recruitment and inflammatory killing	Potential interference with systemic C5a-mediated immune surveillance	Survival and persistence. PPAD suppresses bactericidal mechanisms (complement, phagocytosis, NETs, and antimicrobial peptides) to secure a replicative niche for *P. gingivalis* within the periodontal pocket	(Bielecka et al., 2014)
	Neutrophil phagocytic function	Restricts bacterial binding, internalization, and intracellular killing by neutrophils	Reduces bacterial clearance, enabling persistent subgingival infection	Facilitates OMV/PPAD survival during transient bacteremia and hematogenous dissemination	–	(Gabarrini et al., 2018b)
	NET-associated histone H3	Citrullination of extracellular histone H3 weakens NET structure and reduces bactericidal activity	Promotes bacterial evasion from NET-mediated killing	Released citrullinated histone H3 (CitH3) may serve as a systemic neoantigen in autoimmune priming	–	(Vitkov et al., 2018)
	Antimicrobial peptide LP9	Citrullination abrogates the membrane-disrupting capacity of this lysozyme-derived cationic peptide	Neutralizes a key innate immune effector molecule	–	–	(Stobernack et al., 2018)
ROS modulation	Neutrophil oxidative burst/survival pathways	Suppression of ROS release; upregulation of anti-apoptotic proteins A1/Bfl-1 and Bcl-xL	Reduces oxidative killing; paradoxically prolongs neutrophil lifespan within periodontal lesions	Preserves OMV lipid bilayer integrity and PPAD catalytic competence during systemic transit; protects PPAD from oxidative inactivation	Carrier protection. ROS reduction protects not only the bacterium but also OMV-encapsulated PPAD, ensuring its enzymatic activity upon arrival at distant target organs	(Gawron et al., 2014; Kriebel et al., 2018)
Pro-inflammatory amplification	TLR2/fimbrial components (FimA)	PPAD activity and FimA are prerequisites for TLR2-dependent signaling hyperactivation, upregulating TNF-α, IL-1β, COX-2, and mPGES-1	Establishes a Th17-biased inflammatory microenvironment; drives PGE₂-mediated alveolar bone resorption	Establishes analogous inflammatory milieu in synovium and vascular walls, promoting tissue destruction and immune cell infiltration	Niche remodeling. While suppressing killing, PPAD simultaneously remodels the microenvironment into a chronic inflammatory state (Th17/IL-17 axis, cytokine networks) that favors anaerobic bacterial survival and perpetuates local and systemic inflammation	(Wielento et al., 2022)
	IL-36γ/CXCL8/CCL20	Robust induction of IL-36γ in oral epithelial cells, which amplifies CXCL8 and CCL20 expression	Orchestrates neutrophil and T-cell recruitment; generates a hallmark Th17-biased chronic periodontitis lesion	Similar chemokine cascades may be activated in extra-oral mucosal and synovial tissues	–	(Huynh et al., 2016; Aliko et al., 2018)
	Monocytes/macrophages	Upregulation of TNF-α, IL-1β, IL-6, IL-10, and MCP-1; marked downregulation in PPAD-deficient strains	Amplifies local inflammatory response and osteoclastogenesis	Drives macrophage activation within atherosclerotic plaques and inflamed synovium	–	(Karkowska-Kuleta et al., 2018)
	Gingival fibroblasts	Citrullination of bacterial surface proteins activates the PGE₂ signaling pathway (COX-2, mPGES-1)	Induces local bone resorption, tissue destruction, and delayed wound healing	PGE₂ promotes systemic inflammatory bone loss and vascular inflammation	–	(Gawron et al., 2014)
Tissue barrier disruption	Epidermal growth factor (EGF)	Citrullination impairs EGF function; suppression of SOCS3 and IRF1 signaling inhibits epithelial cell proliferation and migration	Compromises the protective gingival epithelial barrier; delays periodontal tissue healing	Weakened mucosal barriers facilitate bacterial translocation and systemic entry of citrullinated antigens	Gateway opening. Barrier dysfunction at both local (gingival) and systemic (intestinal) levels creates entry portals for OMVs and citrullinated antigens	(Pyrc et al., 2013)
	Intestinal tight junction proteins (occludin, ZO-1)	Downregulation of HNRNPA2B1 suppresses tight junction transcription	–	Disrupts intestinal epithelial integrity, promoting oral–gut axis translocation and IBD exacerbation	–	(Zhang, 2023)
Immune tolerance breakdown	RA-associated autoantigens (fibrinogen, vimentin, α-enolase, collagen II)	C-terminal arginine citrullination generates novel citrullinated epitopes; synergizes with Rgp to produce multiple citrullinated peptides	Local generation of citrullinated proteins that enter circulation via mucosal breaches or OMVs	In synovium, citrullinated proteins act as heterologous antigens inducing cross-reactive ACPAs through molecular mimicry; perpetuates autoimmune arthritis	Systemic autoimmunity. The “safe space” created by immune evasion allows sustained citrullination. OMVs deliver these neoepitopes to distant organs, where they break B-cell tolerance and establish self-sustaining autoimmune memory	(Wegner et al., 2010; Kuna, 2012; Montgomery et al., 2016; Jenning et al., 2020)
	CD4⁺ T-cell differentiation	Promotion of Th17 differentiation and IL-17 expression; suppression of Treg proportion and IL-10 secretion	Sustains chronic periodontal inflammation through pro-inflammatory/anti-inflammatory imbalance	Th17/Treg imbalance in synovium and intestinal mucosa drives RA and IBD pathogenesis	–	(Zhao et al., 2021; Gao, 2022)
	Bradykinin/vascular matrix proteins	Citrullination of bradykinin prolongs vasodilation and increases vascular permeability; citrullination of fibrinogen and vimentin	–	Enhances vascular permeability for inflammatory cell recruitment; citrullinated fibrinogen deposits in atherosclerotic plaques and correlates with coronary artery calcification	–	(Imamura et al., 1995; McGraw et al., 1999; Kuna, 2012; Sokolove et al., 2013)

PPAD, *Porphyromonas gingivalis* peptidylarginine deiminase; OMV, outer membrane vesicle; NET, neutrophil extracellular trap; CitH3, citrullinated histone H3; ROS, reactive oxygen species; TLR2, Toll-like receptor 2; FimA, fimbriae A; COX-2, cyclooxygenase-2; mPGES-1, microsomal prostaglandin E_2_ synthase-1; PGE₂, prostaglandin E_2_; IL, interleukin; TNF-α, tumor necrosis factor-alpha; MCP-1, monocyte chemoattractant protein-1; C5aR, C5a receptor; SOCS3, suppressor of cytokine signaling 3; IRF1, interferon regulatory factor 1; HNRNPA2B1, heterogeneous nuclear ribonucleoprotein A2/B1; ZO-1, zonula occludens-1; Th17, T helper 17 cell; Treg, regulatory T cell; ACPA, anti-citrullinated protein antibody; RA, rheumatoid arthritis; IBD, inflammatory bowel disease; Rgp, arginine-specific gingipain.

The mechanisms summarized in this table are supported by evidence cited in the main text (see sections on local and systemic pathogenic mechanisms for primary references).

## 4 Systemic pathogenic mechanisms of PPAD: from periodontal tissues to distant organs

### 4.1 OMV-mediated systemic dissemination

PPAD can utilize OMVs to breach the local periodontal barrier and achieve systemic dissemination. This “Trojan horse”-like delivery system (Jäger et al., 2015) involves four key steps: PPAD is enriched in OMVs following A-LPS modification (Gabarrini et al., 2018a); the vesicles, owing to their nanoscale size, traverse the damaged gingival epithelium and enter the bloodstream (Wu et al., 2025); subsequently, OMVs are taken up by endothelial cells and macrophages via lipid raft-dependent endocytosis, and their carried PAMPs activate pattern recognition receptors such as TLR2/NOD (Wu et al., 2025); upon internalization, the released PPAD catalyzes the citrullination of host proteins in target organs, breaking immune tolerance and triggering systemic autoimmune inflammation.

The recently identified high-activity PPAD-T2 variant exhibits two-fold higher enzymatic activity *in vitro*, suggesting that it may exacerbate local citrullination burden and enhance pro-inflammatory potential; however, whether it directly increases OMV production or distal dissemination efficiency remains to be validated (Bereta et al., 2019). Overall, by promoting neutrophil survival and synergizing with gingipains to disrupt the epithelial barrier, PPAD is significantly associated with the accumulation of abnormally citrullinated proteins in multiple distal organs, including joints and vascular walls, and is therefore considered a key target for blocking the “local-to-remote” inflammatory axis.

### 4.2 PPAD and rheumatoid arthritis

Rheumatoid arthritis (RA) is a complex, multifactorial autoimmune disease affecting 0.5% to 1% of the world’s population. It is characterized by chronic inflammation of the synovial joints and bone erosion, leading to joint destruction, pain, disability, and reduced life expectancy in untreated individuals (Nishimura et al., 2007). ACPAs and rheumatoid factor are common autoantibodies found in patients with RA (Nishimura et al., 2007). ACPAs, including anti-cyclic citrullinated peptide antibodies as a commonly used subset, are common autoantibodies found in patients with RA (Liu et al., 2022). ACPAs can be detected years before the onset of clinical symptoms and are strongly associated with disease severity, joint damage, and poor prognosis (Lira-Junior and Figueredo, 2016).

PPAD can citrullinate type II collagen, vimentin, α-enolase, and fibrinogen—all known autoantigens in RA—and these citrullinated proteins are targeted by ACPAs, thereby promoting the development of RA (Kuna, 2012). Animal studies have shown that wild-type *P. gingivalis* exacerbates collagen-induced arthritis, whereas a PPAD gene-deficient strain significantly attenuates this effect, and the complementation strain restores the phenotype, indicating that PPAD plays an important role in periodontal infection-driven arthritis progression (Maresz et al., 2013; Gully et al., 2014). However, findings are not entirely consistent across different models and host genetic backgrounds. For instance, in HLA-DRβ1 transgenic C58BL/6 mice, exposure to *P. gingivalis* induced ACPA production and antoimmune arthritis, whereas wild-type C57BL6 mice failed to generate ACPAs under the same conditions, indicating that the HLA-DRB1 shared epitope is required for PPAD-driven ACPA generation (Sandal et al., 2016). Some studies have found that anti-PPAD antibodies are not associated with RA disease severity or with ACPAs (Konig et al., 2015). Other studies have reported that lower levels of anti-PPAD antibodies correlate with better treatment outcomes and a more pronounced reduction in ACPA levels (Kobayashi et al., 2016), suggesting that PPAD may act more as an “amplification factor” rather than a sole trigger (Eriksson et al., 2016).

Castillo et al. enrolled 255 Colombian subjects and demonstrated that anti-RgpA/anti-PPAD double positivity was significantly associated with a diagnosis of RA (OR = 6.63, 95% CI 1.61–27), exhibiting a specificity of 93.7% that was markedly superior to single-marker detection (Castillo et al., 2023). These findings suggest that the humoral immune response directed against two key virulence factors of *P. gingivalis* may be linked to RA, and double antibody positivity could serve as an auxiliary serological marker for screening RA associated with periodontal infection (Castillo et al., 2023). PPAD-mediated citrullination of human proteins may represent the mechanistic bridge connecting periodontitis with RA (Wegner et al., 2010; de Smit et al., 2011). Repeated oral mucosal breakdown associated with chronic periodontitis enables extensively citrullinated bacterial and host proteins within the oral cavity to be released into the bloodstream. This process is closely related to the expansion of inflammatory monocyte subsets in the inflamed synovium and peripheral blood of RA patients and may activate ACPA-positive B cells, thereby promoting epitope spreading (Brewer et al., 2023). Transient bacteremia induced by routine activities such as toothbrushing may further facilitate the systemic dissemination of oral-derived citrullinated antigens (Vitkov et al., 2018). Notably, oral protein citrullination is catalyzed not only by the PPAD of *P. gingivalis* but also by human PADs. These orally derived citrullinated antigens enter the systemic circulation through mucosal breaches, potentially breaking immune tolerance and driving the production of ACPAs, thereby propagating or perpetuating the autoimmune response in RA (Potempa et al., 2017). Castellar-Mendoza et al. reported that although *Porphyromonas gulae* carries a PPAD with up to 93% homology to that of *P. gingivalis*, this bacterium is primarily a canine periodontal pathogen and is rarely detected in humans. Consequently, its lack of association with RA disease activity likely reflects limited human ecological relevance rather than an absence of PPAD pathogenic potential, underscoring that PPAD’s contribution to RA is specifically tied to *P. gingivalis* as a human periodontal pathogen (Castellar-Mendoza et al., 2023). This indicates that the pathogenic potential of PPAD depends not merely on enzymatic activity per se but rather on the virulence background of its bacterial host. Although periodontal treatment ameliorates disease severity in RA patients, it fails to improve antibody levels, suggesting that PPAD-induced autoimmune responses establish persistent immunological memory. The association between *P. gingivalis*-associated periodontitis and RA is indisputable; however, the role of PPAD as a direct link between these two diseases remains to be fully elucidated.

At the mechanistic level, PPAD may participate in the onset and progression of RA through multiple pathways. First, PPAD possesses a unique C-terminal arginine citrullination capacity, enabling the generation of novel citrullinated epitopes. These bacterially derived modified peptides may contribute to the breakdown of immune tolerance against self-citrullinated proteins, thereby inducing the production of ACPAs and representing a potential exogenous precipitant for the autoimmune initiation of RA. Second, *P. gingivalis*-derived PPAD-mediated citrullinated proteins may serve as “heterologous citrullinated antigens”, inducing cross-reactive autoantibodies against host self-citrullinated proteins through molecular mimicry, thereby instigating or propagating autoimmune responses. Lundberg et al. demonstrated that *P. gingivalis* anti-citrullinated α-enolase antibodies cross-react with human anti-α-enolase peptide 1 antibodies (Lundberg et al., 2010). In a comparable study, incubation of human α-enolase with *P. gingivalis* gingipains and PPAD yielded 11 C-terminal citrullinated peptides derived from α-enolase; similarly, incubation of fibrinogen with PPAD and gingipains resulted in the generation of 37 C-terminal citrullinated peptides derived from fibrinogen (Montgomery et al., 2016). Notably, a PPAD variant isolated from an RA patient not only retained C-terminal arginine citrullination activity but also exhibited internal arginine citrullination activity against fibrinogen, vimentin, and other major RA autoantigens. Intriguingly, human PAD4 can similarly modify fibrinogen α-chain residues R271 and R547, suggesting that bacterial and human PAD enzymes may generate functionally overlapping citrullinated epitopes on certain substrates (Jenning et al., 2020). This indicates that bacterially derived citrullinated proteins may act as “pseudo-self antigens”, inducing cross-reactive ACPAs, thereby breaking immune tolerance and instigating autoimmune attack (Muñoz-Atienza et al., 2020). Consequently, PPAD, by virtue of its unique C-terminal specificity and its capacity to induce citrullination of endogenous antigens, establishes an independent exogenous pathogenic pathway distinct from the host PAD enzyme regulatory network (Vitkov et al., 2018).

Furthermore, PPAD synergizes with gingipains to activate pro-inflammatory signaling pathways, inducing the release of inflammatory mediators such as PGE_2_, IL-6, and TNF-α, and driving the activation of the Th17/IL-17 axis, thereby establishing an inflammatory microenvironment conducive to the sustained expansion of autoimmunity. PPAD-catalyzed citrullination stimulates human gingival fibroblasts to produce PGE_2_, a potent bone-resorbing inflammatory mediator implicated in the pathogenesis of both chronic periodontitis and RA (Gawron et al., 2014). In collagen-induced arthritis (CIA) rats orally infected with *P. gingivalis*, the proportion of CD4^+^ T cells differentiating toward Th17 was higher, whereas differentiation toward regulatory T cells was reduced, compared with rats infected with the PPAD-knockout mutant; expression of IL-17 in rat ankle joints was significantly increased, accompanied by synovial hyperplasia and inflammatory cell infiltration within the joint cavity. These findings suggest that the local inflammation generated by *P. gingivalis* in the oral cavity can exacerbate joint inflammation in CIA rats, an effect associated with PPAD secreted from the OM, and that the Th17/IL-17 signaling axis may play a critical regulatory role in immune dysregulation and aggravated joint inflammation in CIA rats (Gao, 2022). Citrullinated proteins generated by both PPAD and human PAD can induce immune responses in synovial Th17 cells (Alghamdi and Redwan, 2022), which are mediated by the release of high levels of pro-inflammatory cytokines including IL-1β, IL-6, IL-22, IL-23, and TNF-α. Th17 cells secrete IL-17, which promotes the production of pro-inflammatory cytokines by macrophages and fibroblasts, facilitates immune cell infiltration into joints, induces the expression of matrix metalloproteinases, and plays a pivotal role in osteoclast differentiation and articular bone erosion (Aquino et al., 2017). However, the capacity of PPAD and FimA to co-activate TLR2 and drive PGE_2_ synthesis depends on enzymatic activity rather than the antigenicity of the protein itself, indirectly promoting autoimmune initiation through the establishment of a pro-inflammatory microenvironment (Prucsi et al., 2023). Through its dual roles in aberrant citrullination and inflammatory immune modulation, PPAD may exert important regulatory effects in both the immune initiation and disease progression of RA.

### 4.3 PPAD and atherosclerosis

Atherosclerosis (AS) is a chronic progressive disease characterized by lipid deposition within the vascular wall, fibrous cap formation, and calcification, resulting in vascular stenosis, obstructed blood flow, and a predisposition to complications such as coronary heart disease and myocardial infarction, thereby posing a serious threat to patient survival (Kloska et al., 2020). RA patients carry a substantially elevated risk of cardiovascular morbidity and mortality compared with the general population, with AS being the leading cause of death in this population; this excess cardiovascular burden is driven partly by chronic systemic inflammation and autoimmunity, including citrullination-driven immune responses that may accelerate atherogenesis independently of traditional risk factors (Gabriel, 2008; Yin et al., 2024). Recent studies have revealed that citrullination plays an important role in the pathogenesis of AS. Giles et al. reported that the intensity of anti-citrulline staining in cardiac muscle was 59% higher in RA patients compared with the non-RA group, suggesting an association between systemic citrullination and cardiovascular pathology (Giles et al., 2012). Sokolove et al. observed significantly increased citrullinated proteins—predominantly citrullinated fibrinogen—in atherosclerotic plaques (Sokolove et al., 2013); moreover, RA-related autoantibodies could target these proteins, and levels of anti-citrullinated fibrinogen antibodies were positively correlated with coronary artery calcification scores, suggesting that citrullinated peptide antigens and their immune complexes may contribute to plaque progression. In addition, ACPA positivity is considered an independent risk factor for coronary heart disease even in non-RA individuals and may perpetuate local plaque inflammation and progression through the formation of immune complexes (Sokolove et al., 2013).

PPAD contributes to immune evasion and mediates inflammatory responses. By suppressing neutrophil phagocytic killing capacity and generating citrullinated peptide antigens, PPAD synergistically exacerbates both local and systemic inflammation. Studies have demonstrated that PPAD induces histone H3 citrullination, enabling citrullinated histone H3 to recruit KCNIP3, thereby suppressing B3GAT1 expression and playing a critical role in glycocalyx injury (Yin et al., 2026). PPAD can citrullinate the vasoactive peptide bradykinin, generating citrullinated bradykinin (McGraw et al., 1999). This PPAD-generated citrullinated bradykinin reduces carboxypeptidase-mediated cleavage efficiency, potentially prolonging its vasodilatory effects, increasing vascular permeability, and facilitating bacterial infiltration into the arterial wall and recruitment of inflammatory cells (Imamura et al., 1995). Furthermore, ammonia produced by PPAD catalysis can locally neutralize environmental pH. Since Rgp activity is significantly higher under alkaline conditions (pH∼7.25) compared to acidic conditions, this pH alkalinization enhances Rgp proteolytic activity, which in turn further promotes bradykinin generation, establishing a positive feedback loop (Kozarov et al., 2005). Citrullinated fibrinogen and citrullinated vimentin are associated with coronary artery calcification scores. Upon release into the circulation, these antigens can induce the production of ACPAs, forming antigen–antibody immune complexes that deposit within the vascular wall and activate macrophages and the complement system via Fcγ receptors, thereby exacerbating plaque inflammation and instability. While wild-type *P. gingivalis* has been shown to accelerate atherosclerosis in ApoE-/- mice through oxidative stress, inflammatory responses, and NF-κB activation, studies directly comparing PPAD-knockout *P. gingivalis* with wild-type strains in atherosclerosis models are currently lacking; nevertheless, the causal contribution of PPAD in this process remains to be further elucidated.

### 4.4 PPAD and inflammatory bowel disease

Inflammatory bowel disease (IBD) encompasses a group of complex chronic inflammatory gastrointestinal disorders, primarily including ulcerative colitis (UC) and Crohn’s disease (CD), clinically characterized by abdominal pain, diarrhea, bloody stools, and weight loss. Over recent decades, the global incidence of IBD has increased significantly, with a notable trend toward younger age groups, posing a substantial challenge to public health (Kelsen et al., 2019). Periodontitis has increasingly been recognized as a comorbid factor of IBD, and the mechanisms by which *P. gingivalis* and its virulence factor PPAD contribute to intestinal immune dysregulation have been gradually elucidated.

Oral-derived PPAD or PPAD-containing OMVs may reach the human colon. Notably,16s rRNA gene sequencing has revealed a significant enrichment of *P. gingivalis* in fecal samples from patients with Crohn’s disease compared with healthy controls, and the gut microbiota of IBD patients exhibits greater resemblance to oral bacteria than that of healthy individuals, supporting the ectopic translocation of oral pathogens to the intestine (Wang et al., 2024; Xiang et al., 2024). *P. gingivalis* possesses the unique ability to tolerate acidic environment and bypass gastric barriers, while IBD-associated decrease in gastric acidity and attenuated colonization resistance further facilitate this translocation (Huang et al., 2024).

PPAD disrupts intestinal immune homeostasis by redirecting CD4^+^ T cell differentiation. *In vitro* experiments demonstrated that, following co-culture with conditioned medium from *P. gingivalis* wild-type or PPAD-complemented (comΔppad) strains, the proportion of T helper 17 (Th17) cells and IL-17 protein expression were significantly elevated in mouse splenic T lymphocytes, whereas the proportion of regulatory T (Treg) cells and expression of the anti-inflammatory cytokine IL-10 were markedly reduced. This pro-inflammatory/anti-inflammatory imbalance was further validated in a dextran sulfate sodium (DSS)-induced UC mouse model: compared with the PPAD-deficient strain, intragastric administration of the wild-type or comΔppad strain significantly exacerbated colonic inflammation, as manifested by aggravated weight loss, elevated disease activity index (DAI), shortened colonic length, and increased histological damage index (Zhao et al., 2021).

The balance between Th17 cells and Tregs is critical for maintaining intestinal immune tolerance (Ueno et al., 2013). In UC patients, the number of peripheral blood Th17 cells is positively correlated with disease activity (Ślebioda et al., 2015), whereas Tregs maintain mucosal homeostasis by secreting IL-10 to suppress pro-inflammatory cytokines such as IL-6 and TNF-α (Roers et al., 2004). *P. gingivalis* exacerbates intestinal inflammation in UC through PPAD; the underlying mechanism may involve PPAD-induced conversion of naive CD4^+^ T cells into pro-inflammatory Th17 cells, suppression of anti-inflammatory Treg generation, establishment of a pro-inflammatory microenvironment, and consequent diffuse hemorrhage and ulceration of the colonic mucosa (McGraw et al., 1999).

PPAD compromises the intestinal epithelial barrier through multiple mechanisms. First, PPAD downregulates the expression of the RNA-binding protein HNRNPA2B1, thereby suppressing the transcription of tight junction proteins occludin and zona occludens-1 (ZO-1) and disrupting the physical intestinal barrier. Second, downregulation of HNRNPA2B1 promotes activation of the TLR4 signaling pathway, increasing the secretion of TNF-α and IL-1β, which further compromises barrier integrity (Zhang, 2023). Furthermore, PPAD may directly citrullinate tight junction proteins, altering their charge properties and spatial conformation, ultimately leading to loosening of intercellular junctions.

### 4.5 PPAD and other diseases

Citrullination of arginine residues in proteins and peptides is implicated in multiple pathological processes and may be associated with juvenile systemic lupus erythematosus (jSLE), oral and digestive system cancers, pancreatic cancer, cervical cancer, interstitial lung disease, and other conditions. Figueredo et al. first detected anti-PPAD antibodies in the serum of jSLE patients (Figueredo et al., 2018). Although the overall levels did not differ significantly from healthy controls, a trend toward elevation was observed in the jSLE subgroup with arthritis. All enrolled patients presented with plaque-associated gingivitis and tested positive for *P. gingivalis*; however, since healthy individuals commonly harbor low levels of red complex bacteria, this finding may reflect shared periodontal pathogen exposure rather than an SLE-specific phenomenon. This interpretation is limited by the lack of control groups with periodontitis alone or RA alone (Figueredo et al., 2018). Notably, the same group subsequently observed that anti-PPAD antibody levels paradoxically increased following periodontal treatment, possibly reflecting treatment-induced bacterial antigen release or immune activation (Sete et al., 2021). However, given that anti-PPAD antibody levels in healthy and diseased patients were similar overall, this post-treatment increase should be interpreted with caution, and its clinical significance remains to be determined. This finding is consistent with previous observations that *P. gingivalis* infection is associated with elevated levels of anti-cardiolipin and anti-β2-glycoprotein I antibodies in SLE patients, and that periodontal disease is considered a modifiable risk factor for SLE onset. However, this study was limited by a small sample size and the lack of controls with periodontitis alone or RA alone, precluding discrimination of whether anti-PPAD antibodies reflect shared risk factor exposure or disease-specific mechanisms. The pathogenesis of jSLE involves an imbalance between IL-17A effector cells and regulatory T cells (Crispín and Tsokos, 2010); PPAD may participate in the development of the SLE arthritis phenotype through a similar Th17/Treg imbalance mechanism, though this remains to be validated in larger-scale studies.

PPAD encoded by *P. gingivalis* citrullinates complement C5a, thereby markedly attenuating its chemotactic activity and pro-inflammatory function (Bielecka et al., 2014). Although this finding was initially established in periodontal inflammation models, subsequent studies have detected *P. gingivalis* OMVs in brain tissue in animal models (Poole et al., 2015), raising the hypothesis that PPAD may interfere with C5a-mediated neuroinflammation through this route; nevertheless, quantitative validation of enzymatic activity within the brain remains to be performed. A direct association between *P. gingivalis* and oral–digestive system cancers may also exist. Tumor suppressor gene p53 mutations, particularly arginine mutations in p53, have been detected at high frequency in pancreatic cancer patients, and K-ras arginine mutations can also be identified in this population. *P. gingivalis* DNA is frequently detected in pancreatic cancer tissues, with concomitant enrichment of arginine mutations in p53 and K-ras; the authors proposed that sustained PPAD-mediated deamination may potentially contribute to mutagenesis indirectly-for example, by citrullinating histones or DNA repair proteins, thereby altering chromatin structure or impairing DNA damage response, which could promote base mispairing during replication- but this hypothesis currently lacks validation by mass spectrometry of mutation sites or gene editing, and remains speculative (Öğrendik, 2017). Using dCas9-PPAD fusion protein technology to target the KISS1 promoter region for localized histone citrullination can reactivate KISS1 expression, reduce the viability of cervical cancer cell lines, and inhibit metastasis (Zhang et al., 2024). This suggests that targeted PPAD applications may possess antitumor potential; however, whether bacterial PPAD exerts analogous effects under physiological conditions remains to be elucidated.

PPAD may also be associated with interstitial lung disease (ILD). Jenning et al. reported that anti-citrullinated PPAD peptide antibodies were significantly correlated with parenchymal lung abnormalities at the time of RA diagnosis (Jenning et al., 2020); notably, *P. gingivalis* has been detected in the lungs and lung microbiome of patients with chronic obstructive pulmonary disease and pulmonary infections, including lung abscesses and empyema, supporting the concept of an oral-lung bacterial translocation axis (Shi et al., 2023). However, pulmonary bacterial load and PPAD enzymatic activity remain to be determined. *In vitro* studies have demonstrated that PPAD can citrullinate lung tissue-enriched proteins, such as HSP90 and HARS, suggesting that PPAD may contribute to the pathogenesis of interstitial lung disease through the oral–lung axis. PPAD may also participate in the development and progression of other diseases, although additional evidence is still required.

## 5 Conclusion

As a virulence factor of *P. gingivalis*, PPAD mediates citrullination reactions, serving as a molecular bridge linking this oral condition with various comorbidities. Citrullinated antigens generated locally within the oral cavity can disseminate systemically via OMVs or bacteremia, subsequently participating in disease pathogenesis at distal organs through mechanisms including neoantigen generation, immune evasion, barrier disruption, and inflammatory dysregulation. However, the pathogenic role of PPAD as an exogenous antigen is context-dependent, and its clinical significance must be evaluated in conjunction with host genetic background and microenvironmental characteristics. Future studies are warranted to elucidate the relative contribution of PPAD at different disease stages and to develop precision intervention strategies targeting this enzyme.

Moving forward, targeting PPAD-mediated pathogenic mechanisms represents a promising avenue for the prevention and treatment of periodontitis and systemic diseases. Citrullination constitutes a critical microbial virulence mechanism and a trigger for numerous systemic diseases; consequently, interest in the development of PPAD inhibitors has grown substantially in recent years. Notably, mangiferin and vismiaquinone A isolated from the methanolic leaf extract of Cratoxylum cochinchinense have been reported to inhibit PPAD activity (Tan et al., 2022). However, their *in vivo* efficacy, selectivity, and pharmacokinetic profiles remain to be evaluated. The identification of additional PPAD inhibitors with improved potency and drug-like properties, as well as the elucidation of their precise binding modes through structural biology approaches, holes considerable promise for the development of adjunctive therapeutic agents against periodontitis and other systemic diseases.
